# Effectiveness of Stem Cell Secretomes in the Regeneration and Functional Recovery of Severed Nerves in Patients with Nerve Injuries: A Systematic Review

**DOI:** 10.3390/cells14070492

**Published:** 2025-03-25

**Authors:** Endika Nevado-Sánchez, María Rodríguez-Díaz, Sandra Núñez-Rodríguez, Andrea Bueno-de la Fuente, Raquel de la Fuente-Anuncibay, Vega Villar-Suárez, Jerónimo Javier González-Bernal, Jorge Labrador

**Affiliations:** 1 Reconstructive and Aesthetic Plastic Surgery Service, Hospital Universitario de Burgos, 09006 Burgos, Spain; endineva@hotmail.com; 2 Research Unit, Hospital Universitario de Burgos, 09006 Burgos, Spain; jlabradorg@saludcastillayleon.es; 3 Department of Health Sciences, University of Burgos, 09001 Burgos, Spain; snr1005@alu.ubu.es; 4 Las Huelgas Health Centre (Burgos), 09001 Burgos, Spain; abueno@saludcastillayleon.es; 5 Education Department, University of Burgos, 09006 Burgos, Spain; raquelfa@ubu.es (R.d.l.F.-A.); jejavier@ubu.es (J.J.G.-B.); 6 Department of Surgery, Medicine and Veterinary Anatomy, Institute of Biomedicine (IBIOMED), Faculty of Veterinary Sciences, Campus de Vegazana, University of León, León 24071, Spain; mvvils@unileon.es

**Keywords:** secretomes, stem cells, nerve regeneration, neurotmesis, nerve injuries

## Abstract

The regenerative potential of mesenchymal stem cell (MSC) secretomes in peripheral nerve injuries warrants rigorous evaluation. This systematic review analyzes their effectiveness in preclinical models of neurotmesis, a complete transection of a nerve. Neurophysiological recovery was assessed through nerve conduction velocity (NCV), a measure of the speed at which electrical impulses travel along a nerve. Following PRISMA guidelines, a systematic search was conducted in PubMed, Scopus, Web of Science, and ScienceDirect (last search July 2024). From 640 initially identified studies, 13 met inclusion criteria, encompassing 514 animals (rats). experimental designs published since 2014 in English or Spanish, focusing on MSC secretomes for nerve regeneration. Exclusion criteria included reviews, case reports, and incomplete data. The risk of bias was assessed using Joanna Briggs Institute tools. Results were synthesized narratively, focusing on functional and structural outcomes. The included studies employed various MSC sources, including adipose tissue, olfactory mucosa, and umbilical cord. Nine studies reported enhanced SFI, favoring secretome-treated groups over controls (mean difference +20.5%, *p* < 0.01). Seven studies documented increased NCV, with up to 35% higher conduction velocities in treated groups (*p* < 0.05). Histological outcomes reported in 12 studies showed increased axonal diameter (+25%, *p* < 0.01), myelin sheath thickness (+30%, *p* < 0.05), and Schwann cell proliferation. Limitations of the included evidence include methodological heterogeneity and variability in outcome measurement tools. MSC-derived secretomes demonstrate potential as advanced therapeutic strategies for nerve injuries. Personalized approaches considering injury type and clinical context are essential for optimizing outcomes.

## 1. Introduction

Peripheral nerve injuries, particularly those resulting in neurotmesis [1,2,3], present a highly complex clinical challenge due to the limited intrinsic regenerative capacity of nerve tissue [1,2,4,5,6,7], despite the treatment options available to date [3,4,7,8,9,10,11]. The endogenous reparative response of the peripheral nervous system is insufficient to restore anatomical and functional continuity in cases of neurotmesis, where loss of alignment and Wallerian degeneration in the distal segment severely compromise axonal regeneration [2]. Additionally, factors such as the development of painful neuromas, intraneural fibrosis, and the presence of physical and chemical barriers inhibiting axonal growth further complicate the nerve repair process [12,13,14,15].

The sequelae of these injuries are significant, as they can result in permanent neurological deficits, including paralysis, loss of sensation, and chronic neuropathic pain disorders [16,17]. Despite advances in nerve microsurgery [18], such as epineural neurorrhaphy and the use of autologous grafts, the rate of functional axonal regeneration remains suboptimal [19,20,21,22,23,24,25]. Current surgical procedures face significant limitations, such as mismatched calibers of repaired nerves, disorganization of nerve fibers at the suture site, and the potential formation of perineural scars that inhibit axonal growth [5,26,27]. Although autologous grafts are the gold standard, their use is not without issues, including donor site morbidity, additional surgical time, and challenges in achieving complete and precise reinnervation [28,29].

In this context, there is growing interest in exploring new therapeutic strategies that can overcome these limitations. Among these, mesenchymal stem cell (MSC)-derived secretomes stand out as a promising alternative. MSC secretomes consist of a complex array of bioactive factors, including cytokines, growth factors, and extracellular vesicles, which play a crucial role in modulating the inflammatory response, promoting angiogenesis, and providing neuroprotection [30]. These components have the ability to create a favorable microenvironment for axonal regeneration and functional recovery of severed nerves [31,32,33,34].

Interest in MSC secretomes lies in their ability to offer a less invasive and potentially more effective therapeutic approach compared to conventional strategies [35]. Unlike cellular therapies, which face challenges related to cell viability and differentiation control, secretomes act through the controlled release of bioactive molecules, potentially overcoming some of the limitations associated with direct cell therapy [30,35]. One of the key advantages of secretomes over whole-cell therapies is their lower risk of immune rejection and tumorigenicity, as they do not involve the direct transplantation of living cells. Additionally, secretomes can be standardized, stored, and administered more easily, making them a more practical and scalable therapeutic option. Their acellular nature also allows for better regulatory compliance and reduces concerns associated with cell survival and engraftment.

Given the transformative potential of MSC secretomes in nerve regeneration, it is imperative to rigorously and systematically evaluate their effectiveness. This systematic review aims to analyze the regenerative capacity of mesenchymal stem cell secretomes in preclinical and clinical models of peripheral nerve injuries due to neurotmesis. Our objective is to provide a critical, evidence-based assessment of the role of secretomes in the functional recovery of injured nerves, aiming to delineate their viability as an advanced therapeutic strategy for treating these complex injuries.

## 2. Materials and Methods

Following the guidelines of the PRISMA Statement [36], and in accordance with a previously defined research protocol, a systematic review of the scientific literature was conducted between 1 July and 15 July 2024. The electronic versions of the PubMed, Scopus, Web of Science, and ScienceDirect databases were consulted for this purpose. The search began by formulating a clinically relevant research question in PIO format (Table 1), as proposed by Sackett et al. [37].

Once the research question was formulated, various search strategies were designed and tailored to the specific characteristics of each database. Relevant Medical Subject Headings (MeSH) were used, combined with Boolean operators (AND/OR), along with free-text terms, some of which were truncated to capture all possible keyword variations. The search strategies we followed in the review, adapted to each database used, are detailed below.

1. Pubmed: ((stem cell secretome [Title/Abstract] OR stromal cell secretome [Title/Abstract] OR mesenchymal secretome [Title/Abstract] OR stem cell conditioned medium [Title/Abstract] OR stromal cell conditioned medium [Title/Abstract] OR secretome [Title/Abstract] OR conditioned medium [Title/Abstract]) AND (nerve regeneration [Title/Abstract] OR nerve repair [Title/Abstract] OR nerve healing [Title/Abstract] OR neural regeneration [Title/Abstract]) AND (nerve injury [Title/Abstract] OR nerve lesion [Title/Abstract] OR nerve damage [Title/Abstract] OR nerve transection [Title/Abstract] OR nerve cut [Title/Abstract] OR nerve rupture [Title/Abstract])).
2. Web of Science: TS = ((“stem cell secretome” OR “stromal cell secretome” OR “mesenchymal secretome” OR “stem cell conditioned medium” OR “stromal cell conditioned medium” OR “secretome” OR “conditioned medium”) AND (“nerve regeneration” OR “nerve repair” OR “nerve healing” OR “neural regeneration”) AND (“nerve injury” OR “nerve lesion” OR “nerve damage” OR “nerve transection” OR “nerve cut” OR “nerve rupture”)).
3. Scopus: TITLE-ABS-KEY((“stem cell secretome” OR “stromal cell secretome” OR “mesenchymal secretome” OR “stem cell conditioned medium” OR “stromal cell conditioned medium” OR “secretome” OR “conditioned medium”) AND (“nerve regeneration” OR “nerve repair” OR “nerve healing” OR “neural regeneration”) AND (“nerve injury” OR “nerve lesion” OR “nerve damage” OR “nerve transection” OR “nerve cut” OR “nerve rupture”)).
4. Science Direct: (“stem cell secretome” OR “stromal cell secretome” OR “mesenchymal secretome” OR “stem cell conditioned medium” OR “stromal cell conditioned medium” OR “secretome” OR “conditioned medium”) AND (“nerve regeneration” OR “nerve repair” OR “neural regeneration”) AND (“nerve injury” OR “nerve transection” OR “nerve cut” OR “nerve rupture”).

The review included original studies that met the following criteria: (1) presented an appropriate methodological design to evaluate the effectiveness of stem cell secretomes in the regeneration of severed nerves, (2) published in English or Spanish, (3) published since 2014, (4) had at least an accessible abstract, and (5) provided relevant data on nerve regeneration and functionality in experimental or clinical models of nerve injuries. Excluded were case reports, letters to the editor, low-quality reviews, and studies that did not directly address the research question or focused on specific subgroups of the population, such as patients with preexisting conditions or those with non-severed nerve injuries.

As a complementary strategy, a manual reverse search, also known as “snowballing”, was conducted to identify additional relevant studies that had not been initially considered. Both gray literature and the bibliographic references cited in the selected studies were reviewed.

Study selection and methodological quality assessment were performed independently and blindly by two reviewers with Rayyan software (website: https://www.rayyan.ai/). Discrepancies were resolved through consensus, with the involvement of a third reviewer in cases of persistent disagreement, also with Rayyan software. To ensure consistency among researchers during data collection, a standardized information extraction form was developed. This form included the following elements for each selected article: title and lead author, country and year of publication, study type and objectives, location and publication period, sample size and characteristics, definition of analyzed variables and instruments used, a summary of results and conclusions, and the outcomes of the scientific and technical quality assessment.

To evaluate methodological quality and risk of bias, the Joanna Briggs Institute’s “critical appraisal tools” from the University of Adelaide [38] were used, adapted to the design of each study [39]. An acceptance threshold of at least 9 out of 13 was established for the inclusion of experimental studies in the systematic review. A pilot test was conducted in which each reviewer assessed three articles, followed by an analysis of inter-rater agreement.

For included studies, missing data were addressed by reviewing supplementary sources and contacting authors for additional information. If information could not be retrieved, a sensitivity analysis was applied to determine the impact of missing data on the overall results. In cases where missing data significantly affected the interpretation of the results, studies were excluded from the final analysis.

Table 2 summarizes the characteristics and outcomes of the studies selected for the review.

## 3. Results

**Figure 1:**
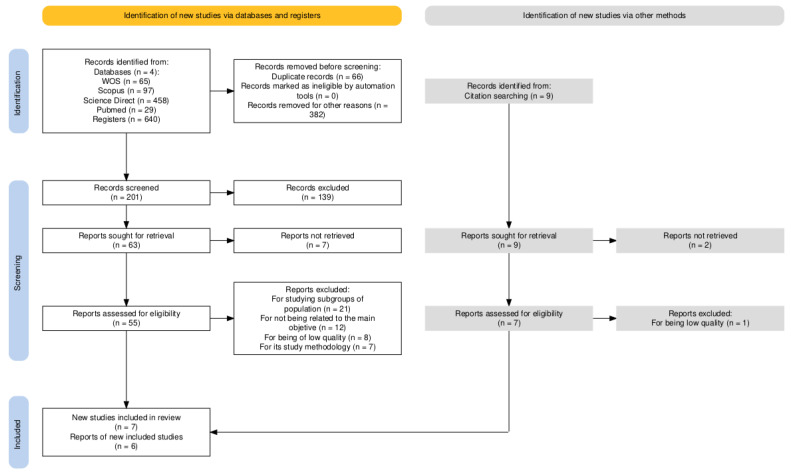
Flow diagram for study selection.

Of the 640 documents initially identified, 14 experimental studies were selected for systematic review following a full-text critical appraisal. The selection process is illustrated in the flow diagram below (Figure 1).

On the other hand, the main data from the selected studies, including their key characteristics and findings, are summarized in Table 2.

### 3.1. Description of Study Characteristics

A total of 13 studies were included in this review, with participant numbers ranging from 20 to 95, encompassing 514 animal models of rats. Among the reviewed studies, 10 used Sprague Dawley (SD) rats, while three employed *Wistar rats*. The age of the animal models varied between 2 and 12 weeks, and the weights ranged from 170 g to 400 g. All studies adopted a longitudinal quantitative experimental design, which allowed for a detailed evaluation of the effectiveness of different therapeutic approaches in nerve regeneration.

The included studies focused on the application of conditioned media and secretomes from various types of mesenchymal stem cells, including those derived from adipose tissue, olfactory mucosa, and the umbilical cord, to promote nerve regeneration in animal models of injury. Through various methodologies, aspects such as motor functionality, electrophysiological recovery, and histological characteristics of regenerated nervous tissue were explored. This review highlights how these approaches not only favor structural regeneration but also contribute to the restoration of sensory and motor functions, thus providing a comprehensive overview of the effectiveness of MSC secretomes in regenerative medicine.

The studies evaluated variables related to nerve functionality and regeneration using various tools. Paw print analysis was employed in seven studies to assess motor function using different methods, including the Sciatic Functional Index (SFI), which was calculated in six studies from specific measurements of paw prints. Additionally, electrophysiological tests, such as nerve conduction velocity (NCV), were used in seven investigations, providing a comprehensive understanding of motor performance and nerve activity.

The histological characteristics of regenerated nervous tissue were examined through staining techniques like hematoxylin and eosin (H&E), used in eight studies, as well as immunohistochemical staining for myelination markers and transmission electron microscopy (TEM) in five studies, allowing for a detailed evaluation of cellular morphology. Moreover, four studies conducted cell viability assays and cytokine analysis to explore the biological responses to secretome treatment.

The statistical methods used in the studies varied depending on the specific objectives of each study, but most used SPSS software (versions 17.0 and 21.0) and GraphPad Prism (versions 5.0 and 8.0) for data analysis. Unidirectional and bidirectional analysis of variance (ANOVA) were applied to assess differences between multiple groups, complemented with Tukey or Bonferroni post hoc tests to determine significant comparisons. Data were generally expressed as means ± standard deviation (SD) or means ± standard error of the mean (SEM), ensuring proper presentation of the results. Additionally, a *p*-value of <0.05, and in some cases < 0.01, was considered indicative of statistical significance, allowing researchers to establish solid conclusions from the observed differences in their experiments.

In analyzing the methodological quality and risk of bias of the studies (Table 3), most obtained high to medium scores, always exceeding the established cutoff.

### 3.2. Description of the Results

This systematic review has allowed for the identification of significant findings that highlight the capacity of these treatments to improve the functional and structural recovery of the nervous system. All the studies analyzed show a consensus that secretomes derived from different types of stem cells contribute positively to nerve regeneration [40,41,42,43,44,45,46,47,48,49,50,51,52], manifesting improvements in various functional and histological parameters.

The studies included in this review employed different types of secretomes. The subcutaneous papilla dermal stem cell secretomes (SKP-SC) [40] and their exosomes [42] showed a significant increase in TGT regeneration scores (*p* < 0.001). They also enhanced the expression of nerve regeneration markers, such as *NF200* and *S100β*. Additionally, these secretomes contributed to a larger diameter of myelinated nerve fibers and an increase in myelin sheath thickness (*p* < 0.001). They also improved motor neuron survival rates, reaching 90% (*p* < 0.001).

The adipocyte-derived mesenchymal stem cells (ADSCs) [41,52] showed a dose-dependent effect in improving stem cell viability when co-cultured with SKP-SC exosomes. They also promoted an increase in the length and number of motoneuron axons (*p* < 0.01). These findings suggest a beneficial impact on the functional and electrical recovery of regenerating nerves (*p* < 0.001).

The *NGF*-stimulated adipocyte-derived stem cells *(STM-NGF-ASC*) [43] demonstrated significant promotion of axonal growth in in vitro studies. The conditioned media from stem cells (CM) [44,45,46,53] have been associated with better functional recovery and a significant improvement in nerve regeneration compared to control groups.

The stem cell-derived exosomes [47,48,49,50,51] proved effective in promoting myelination and axon regeneration in sciatic nerves treated with ASC-Exos. This suggests a positive mechanism of action in nerve recovery.

Finally, the gingival tissue-derived stem cell exosomes (GMSCs) [49] showed promising results. These exosomes enhanced Schwann cell proliferation and improved myelination.

Nerve functionality assessed with the Sciatic Functional Index (SFI) in nine studies from the review [41,42,43,44,45,46,47,49,51] showed significant improvements in the scores of groups treated with stem cell secretomes. Chen et al. reported a significant increase in the mean SFI score at 6 weeks post-surgery in rats treated with SKP-SC (*p* < 0.001) [40]. Similarly, Fu et al. [41] found that adipocyte-derived mesenchymal stem cells (ADSCs) generated a notable increase in SFI (*p* < 0.01). In Yu et al.’s study [42], co-cultivation with SKP-SC-EV significantly improved motor neuron survival, resulting in an SFI improvement at 12 weeks post-surgery. On the other hand, one study evaluated nociceptive function using the withdrawal reflex latency (WRL) test and motor deficit using the extensor postural thrust (EPT), associating it with significant improvement in the experimental group compared to the control (*p* < 0.001) starting from the 3rd week after treatment with UCX cells [52].

Furthermore, the studies highlighted significant improvements in the electrical function of the regenerated nerve through compound muscle action potential (CMAP) amplitude, showing that interventions with stem cell secretomes and biomaterials significantly improved functional recovery compared to control groups [41,42,46,47]. In particular, increases in CMAP amplitude and nerve conduction velocity were observed; also, recovery in the wet weight ratio of target muscles was observed [40,44,47,51], suggesting that secretome treatments also contribute to better muscle recovery after denervation.

The diameter of myelinated nerve fibers and the thickness of the myelin sheath also showed notable improvements [40,41,42,44,47]. Prautsch et al. [43] reported robust axonal regeneration and larger fiber diameter in rats treated with *NGF*-stimulated ASC [43]. Additionally, Alvites et al.’s [44] findings indicated that the CMOM group had a lower motor deficit and better SFI compared to the End-to-End group (*p* = 0.0016), along with reduced muscle mass loss [44].

Complementing these findings, histological findings indicate that treatments with stem cells and stem cell-derived exosomes significantly improve nerve regeneration, evidenced by an increase in myelination, axon diameter, and Schwann cell proliferation [40,41,43,44,45,46,47,48,49,50,51,52]. Furthermore, better functional recovery was observed in treated groups compared to controls, as well as increased neurotrophic marker expression and a reduction in inflammation at the injury site. In certain studies, a significant increase in axonal diameter was observed [40,44,47,51,52], suggesting an improvement in the quality of regenerated fibers.

In summary, analyzing the effectiveness of different secretomes in nerve regeneration shows that subcutaneous papilla dermal stem cell secretomes (SKP-SC) are particularly effective for acute injuries, as evidenced by significant increases in TGT regeneration scores and motor neuron survival, reaching up to 90% (*p* < 0.001) [40,42]. These results contrast with adipocyte-derived mesenchymal stem cell secretomes (ADSC), which, although demonstrating an improvement in axon length and number (*p* < 0.01), are more suitable for injuries requiring a long-term functional recovery approach due to their ability to promote axonal growth [41,43,52]. On the other hand, stem cell-derived exosomes were shown to be effective in myelination and axon regeneration in sciatic nerves, making them preferable for peripheral nerve injuries [48,49,50,51]. Conditioned media from stem cells (CM) stood out in inflammatory contexts, where their ability to improve myelination and functional recovery is crucial [45,46]. Lastly, *NGF*-stimulated adipocyte-derived stem cell secretomes (*STM-NGF-ASC*) are particularly promising for in vitro axonal growth, making them relevant in severe axonal injury cases [43]. In conclusion, while all analyzed secretomes show benefits in nerve regeneration, their effectiveness varies according to the type of injury and clinical context, suggesting the need for a personalized approach in regenerative therapy.

## 4. Discussion

Peripheral nerve regeneration in a preclinical rat model of neurotmesis remains a major challenge. Secretomes derived from mesenchymal stem cells (MSCs) have emerged as a promising strategy to improve both functional and structural recovery of injured nerves [30,33,35]. This systematic review evaluated their regenerative capacity, confirming their potential to optimize nerve repair.

Findings indicate that that subcutaneous dermal papilla stem cell secretomes (SKP-SC) [40,42] play a key role in nerve regeneration. These secretomes significantly improve TGT regeneration scores and neuronal viability, suggesting a potent mechanism for acute nerve repair. Meanwhile, adipocyte-derived mesenchymal stem cell secretomes (ADSC) [41,52] contribute to nerve recovery by increasing axon length and number (*p* < 0.01). Their dose-dependent effect suggests that ADSC secretomes may be particularly effective in long-term recovery scenarios.

The use of MSC-derived exosomes has shown promising results in myelination and axon regeneration in peripheral nerves [18,47,48,49,50]. Given that proper myelination is crucial for functional recovery, these exosomes represent a valuable therapeutic approach. *NGF*-stimulated adipocyte-derived stem cells (*STM-NGF-ASC*) [43] also promote axonal growth in vitro, highlighting their potential application in severe injuries.

Additionally, conditioned media (CM) from MSCs [44,45,46,53] have demonstrated benefits in inflammatory environments by improving myelination and functional recovery. Secretomes from adipose-derived (ADSC), olfactory mucosa (OM-MSC), and umbilical cord (UC-MSC) stem cells suggest that the biological context of the injury significantly influences treatment effectiveness. This underscores the need to tailor therapies based on injury type and inflammatory conditions.

Despite the advances made, there are research gaps that need to be addressed in future studies, and these results should be interpreted while taking into account the limitations of the review, such as the lack of assessment of publication bias or the lack of meta-analysis. It is essential to conduct randomized controlled clinical trials evaluating the safety and efficacy of secretome treatments in human populations with peripheral nerve injuries. These studies should include long-term follow-up to determine the sustainability of the observed benefits.

Moreover, it is suggested that the underlying molecular mechanisms that allow for nerve regeneration mediated by secretomes are investigated, which could facilitate the optimization of therapies and the identification of predictive biomarkers of treatment response. Evaluating the effectiveness of different combinations of secretomes and their application in specific contexts, such as acute versus chronic injuries, should also be a focus of future research.

Nevertheless, the findings have important clinical implications. Evidence suggests that MSC-derived secretomes could become a viable therapeutic option for improving functional recovery in peripheral nerve injuries. Their integration into clinical protocols could enhance patient outcomes and overall quality of life.

Personalized regenerative treatments tailored to injury type and patient context may further optimize clinical outcomes. Additionally, health policies could promote further research and clinical implementation of secretome-based therapies, fostering collaborations between research institutions and healthcare providers.

This review has several strengths. It compiles a broad selection of high-quality studies on the efficacy of MSC-derived secretomes, providing a comprehensive evaluation of their therapeutic potential. Moreover, its rigorous methodology enhances the validity and reliability of the findings while highlighting key action mechanisms.

However, there are still gaps in research that future studies should address. Randomized controlled clinical trials are needed to evaluate the safety and efficacy of secretome treatments in human populations with peripheral nerve injuries. These studies should include long-term follow-ups to determine the sustainability of observed benefits. A meta-analysis would be an excellent approach to synthesize and quantify the findings from the various studies included in this review.

Further investigations into the molecular mechanisms underlying secretome-induced nerve regeneration could lead to therapy optimization and the identification of predictive biomarkers. Additionally, future research should explore the effectiveness of different secretome combinations in acute vs. chronic injuries.

Finally, evaluating the feasibility of large-scale secretome production and standardization in clinical settings is crucial for overcoming barriers to widespread implementation. Addressing these aspects could accelerate the integration of secretome-based therapies into mainstream clinical practice.

## 5. Conclusions

The present systematic review highlights the potential of mesenchymal stem cell-derived secretomes as a promising strategy for peripheral nerve regeneration in patients with nerve injuries. Findings suggest that different types of secretomes, such as those derived from subcutaneous dermal papilla stem cells and adipocyte-derived mesenchymal stem cells, offer significant benefits in improving functional and structural parameters, such as motor neuron viability and myelination of nerve fibers. Further studies are recommended to optimize treatment protocols and explore the combined use of different types of secretomes to maximize functional recovery in patients with nerve injuries. These actions will not only contribute to improving clinical outcomes but could also establish new guidelines in the management of peripheral nerve injuries.

## Figures and Tables

**Table 1:** PIO format.

Population	Patients with nerve injuries (nerve sections caused by cuts, ruptures, or other circumstances).
Intervention	Use of stem cell secretomes.
Results	Regeneration and functionality of severed nerves.
Research Question	What is the effectiveness of stem cell secretomes in the regeneration of severed nerves in patients with nerve injuries?

**Table 2:** Characteristics of the studies included in the systematic review.

Study/Author	Study Type/Objective	Participants	Variables/Instruments	Main Findings	JBI
Chen et al. [40], 2024	Design: Longitudinal quantitative. Objective: To explore the effectiveness of repair and potential mechanisms of skin precursor-derived Schwann cell (SKP-SC) implantation in rat brachial plexus injury (BPI) combined with post-neurotomy neurorrhaphy.	N = 30 Species: *Sprague Dawley rats* (SD) Age: 8 weeks Sex: Male Weight: 220–240 g	Motor Function: Terzis Grooming Test (TGT). Cold Sensitivity: Acetone evaporation test. Electrophysiological Assessment: Compound muscle action potential (CMAP) recording. Nerve Morphology Observation: Immunohistochemical staining and confocal microscopy. Morphometric Analysis of Muscles: Cross-sectional sections and antibody staining. Neuronal Cell Viability: CCK8 Tetrazolium Salt Reduction Assay. Secreted Cytokines: Rat Cytokine Array GS67.	Rats treated with SKP-SC showed a significant increase in TGT scores, reaching a score of 5 in two rats at 6 weeks post-surgery, whereas no rats in the PBS group reached this level (*p* < 0.001). The expression of *NF200* and *S100β* in the SKP-SC group was significantly higher compared to the PBS group, indicating increased nerve fiber regeneration and Schwann cell proliferation (*p* < 0.01). The diameter of myelinated nerve fibers in the SKP-SC group showed a significant increase compared to the PBS group (*p* < 0.001), and the thickness of the myelin sheath was also greater in the SKP-SC group (*p* < 0.01). Treatment with SKP-SC-CM increased neuronal cell viability to approximately 90% in the 20% SKP-SC-CM-treated group compared to 60% in the Oxygen-Glucose-Deprivation (OGD) group (*p* < 0.001). Thirty-two pro-regenerative cytokines were identified in the SKP-SC conditioned medium, including factors related to inhibition of apoptosis and promotion of neurogenesis.	10/13
Fu et al. [41], 2019	Design: Longitudinal experimental quantitative. Objective: To evaluate the regenerative capacity of mesenchymal stem cells derived from adipose tissue (ADSCs) to regenerate nerves in a rat sciatic nerve injury model.	N = 95 Sex: Male Species: *Wistar rats* Weight: 100–220 g	Nerve Regenerative Function: Walking track analysis. Nerve Conduction Velocity (NCV): NCV and wave amplitude (WA) analysis through electrophysiological examinations using a biological function testing system. Structure of Regenerated Nerve: Transmission electron microscopy (TEM). Motor Endplate Activation: Acetylcholinesterase (*AchE*) staining. Retrograde Neural Tracing: Neural tracing using horseradish peroxidase (HRP). Gene Expression: Real-time PCR (RT-qPCR). Specific Proteins: Western blot analysis.	Adipose-derived mesenchymal stem cells (ADSCs) were successfully differentiated into Schwann cell-like cells, showing positive expression of *GFAP* and *S100* markers, indicating their ability to adopt nerve support cell characteristics. The Schwann cell-like cell treatment group (SC-L) exhibited a significant increase in SFI, reaching a value higher than the Model group (*p* < 0.01), indicating improved regenerated nerve function. In the SC-L group, there was a significant increase in NCV and wave amplitude (WA) compared to the ADSC and Dulbecco’s Modified Eagle Medium (DMEM) groups, with statistically significant differences (*p* < 0.01). Regenerated nerve fibers in the SC-L group displayed a more complete and uniform myelin sheath structure, similar to that of the control group. In the SC-L group, levels of Brain-Derived Neurotrophic Factor (*BDNF*), Ciliary Neurotrophic Factor (*CNTF*), and Nerve Growth Factor (*NGF*) were significantly higher compared to the Model, ADSC, and DMEM groups (*p* < 0.01).	13/13
Yu et al. [42], 2021	Design: Longitudinal experimental quantitative. Objective: To generate an artificial nerve graft incorporated with extracellular vesicles derived from skin precursor-derived Schwann cells (SKP-SC-EV) to examine the in vivo effects of SKP-SC-EV on peripheral nerve regeneration.	N = 50 Sex: Male Species: *Sprague Dawley (SD) rats* Weight: 80–250 g Age: 10 young/40 adults	Peripheral Nerve Repair: Behavioral analysis, walking track tests, electrophysiological evaluation. SC and MN Cell Viability: Cell Counting Kit-8 (CCK-8). Axon Growth in MN: ImageJ software (Version 1.54p). Release of SKP-SC-EV: Nanoparticle Tracking Analysis (NTA). Postoperative Motor and Sensory Function: CatWalk XT 9.0 and plantar tests. Histological and Electrophysiological Analysis: Transmission electron microscopy (TEM) and electromyography.	The viability of stem cells significantly increased when co-cultured with SKP-SC-EV, showing a dose-dependent increase at concentrations of 4, 8, 16, and 32 × 10^8^ particles/mL. Axons of motoneurons co-cultured with 4 × 10^8^ particles/mL of SKP-SC-EV showed a significant increase in length and branching number compared to the control group (PBS). At 12 weeks post-surgery, the group that received SKP-SC-EV grafts showed a significantly higher SFI value, indicating better functional recovery. The CMAP amplitude in the EV-NG group was significantly higher than in the NG group, demonstrating better recovery of nerve electrical function. The length of regenerated nerves in the EV-NG group was 1.6 times greater than in the NG group at two weeks post-graft. The wet weight ratio of target muscles in the EV-NG group was significantly greater than in the NG group, suggesting better muscle recovery after denervation.	13/13
Prautsch et al. [43], 2020	Design: Longitudinal quantitative experimental. Objective: To evaluate the capacity of adipose-derived stem cells (*ASC*) in response to exogenous growth factor stimulation (such as *NGF* and *VEGF*) to promote axonal regeneration both in vitro and in vivo.	N = 36 Species: *Sprague Dawley rats* Age: Approximately 12 weeks Weight: 250–300 g Sex: Female	Functional Nerve Regeneration: Sciatic Functional Index (SFI). Histological Nerve Regeneration: Hematoxylin and eosin (H&E) staining and immunohistochemistry for *S100* (Schwann cell specific protein). Nerve Fiber Density and Vascularization: Immunohistochemistry for specific markers (NF-200 for nerve fibers and *CD31* for blood vessels). Presence of Fibrosis: Masson’s trichrome staining.	*NGF*-stimulated ASC secretome (*STM-NGF-ASC*) promoted significant axonal growth in vitro, achieving an axonal length of 657 ± 224 μm and an area of 1.76 ± 0.65 mm^2^, compared to unstimulated ASC (80 ± 56 μm in length and 0.083 ± 0.039 mm^2^ in area). In vivo, treating a 10 mm sciatic nerve injury with intramural administration of *NGF-stimulated ASC* (*FNC-W(NGF-ASC))* resulted in robust axonal regeneration in the mid-nerve section, with 6190 ± 2061 axons/mm^2^, compared to 3075 ± 1432 axons/mm^2^ in the intraluminal administration group *(FNC-L(NGF-ASC)).* The intramural administration of *NGF*-stimulated *ASC* was more effective in promoting axonal regeneration compared to other administration methods or unstimulated *ASC*, being superior both in the mid and distal regions of the regenerated nerve.	9/13
Alvites et al. [44], 2022	Design: Longitudinal quantitative experimental. Objective: To evaluate the effectiveness of the conditioned medium (CM) obtained from olfactory mucosa-derived mesenchymal stem cells (OM-MSCs) in combination with nerve conduits (Reaxon^®^ NGCs) to enhance the histomorphometric and functional regeneration of the sciatic nerve in rats after neurotmesis injury.	N = 30 Age: 8–9 weeks Weight: 250–300 g Sex: Males Species: *Sprague Dawley rats*	Nerve Regeneration: Light and electron microscopy, histomorphometric analysis. Motor Performance: Extensor postural thrust (EPT) test. Pain Sensitivity: Withdrawal reflex latency (WRL). Gait Pattern: Sciatic Functional Index (SFI) and static sciatic index (SSI). Muscle Atrophy: Histopathological analysis (H&E) and muscle fiber diameter measurements.	At 20 weeks, the CMOM group showed significantly less motor deficit compared to the UC group, although there were no significant differences among the therapeutic groups (*p* < 0.0001). At 20 weeks, the CMOM group recorded the lowest withdrawal reflex latency time, indicating better recovery, but with no significant differences among other groups. At the end of the study, the CMOM group had the best SFI, significantly outperforming the EtE group (*p* = 0.0016). The EtE group had the highest number of regenerated nerve fibers, totaling 17,423 ± 2217 fibers. The CMOM group had the largest axonal diameter recorded, at 2.862 ± 0.188 μm, indicating better regeneration. The CMOM group showed only a 29.14 ± 7.06% muscle mass loss, in contrast to the ECMOEC group, which had a loss of 57.84 ± 14.53%. The CMOM group exhibited a muscle fiber area of 2700.57 ± 632.7 μm^2^, comparable to the UC group values but significantly different from the ECMOEC and CMOEC groups (*p* < 0.0001).	11/13
Margiana et al. [45], 2019	Design: Longitudinal quantitative experimental. Objective: To evaluate the effectiveness of conditioned medium derived from mesenchymal stem cells (UC-MSC) in sciatic nerve regeneration in an animal model of nerve injury, analyzing its impact on motor functionality, electrophysiological response, and histological characteristics at different post-injury intervals.	N = 54 Age: 2–3 months Weight: 250–300 g Species: *Sprague Dawley rats*	Motor Function: Sciatic Functional Index (SFI), Toe Function Index (TFI), Plantar Functional Index (PFI), and step analysis (Q1, Q2, Q3, Q4, TOA). Electrophysiological Analysis: Measurement of minimum and maximum stimuli, conduction velocity, and nerve conduction time. Histological Analysis: Evaluation of connective tissue, myelin sheath, blood vessels, and Schwann cells in histological sections.	The CM-treated group showed functional recovery at 14 days post-injury (dPI), whereas the standard therapy group (TS) only showed repair at 28 dPI. In the CM group, footprint formation (Q1–Q4 and TOA) was observed from 7 dPI, whereas in the TS group, footprint formation was not observed until 28 dPI. Conduction velocity in the CM group was similar to the sham group, and significantly superior to the TS group at 7 and 70 dPI. The CM group had a minimal stimulus of 40–60 mV, lower than that of the TS group (>70 mV), indicating higher electrical sensitivity in the CM group. Additionally, the CM group showed a higher maximal stimulus than the TS group. The CM and sham groups had a shorter latency time to reach the compound action potential compared to the TS group at 7 and 70 dPI, indicating a better response to stimulation. The CM group presented a larger average diameter of the myelin sheath compared to the standard and sham groups at 7 dPI. The expression of *S100* was higher in the CM group than in the TS group, suggesting better development of Schwann cells in the conditioned medium-treated group.	9/13
Raoofi et al. [46], 2021	Design: Experimental longitudinal quantitative. Objective: To evaluate the effectiveness of a polycaprolactone (PCL) scaffold loaded with conditioned medium from mesenchymal stem cells (CM) in the regeneration of the transected sciatic nerve in *Wistar rats*.	N = 24 Sex: Male Species: *Wistar rats* Weight: Approximately 260 g	Regeneration: Histological evaluations and neurotrophic factor expression tests (RT-PCR). Nerve Functionality: Sciatic Functional Index (SFI) and electromyography. Cytotoxicity: L929 fibroblast cell line. Morphology: Scanning electron microscopy (SEM). Biomarker Analysis: Western blot and RT-PCR.	Rats in the axotomy group showed a significant decrease in SFI values at 12 weeks (*p* < 0.001). The scaffold and scaffold-loaded CM groups displayed less motor deterioration (*p* < 0.01) and better recovery compared to the axotomy group. A reduction in latency was recorded in the scaffold and scaffold-loaded CM groups (*p* < 0.05), indicating an improvement in muscle function. Moreover, CMAP amplitude was higher in the CM-loaded scaffold group compared to the scaffold group (*p* < 0.05). A significant increase in the total number of nerve fibers and the thickness of the myelin sheath was observed in the treated groups compared to the axotomy group (*p* < 0.01), highlighting the effectiveness of mesenchymal stem cells in regeneration. In the axotomy group, the total number of neurons and satellite cells was significantly reduced compared to the scaffold (*p* < 0.05) and CM-loaded scaffold (*p* < 0.01) groups. The expression of the genes *Ngf*, *Bdnf*, and *S100* was significantly reduced in the axotomy group compared to the control (*p* < 0.01), while overexpression was observed in the CM-loaded scaffold group (*p* < 0.01).	11/13
Cong et al. [47], 2025	Design: Experimental longitudinal quantitative. Objective: To identify the main putative contributors associated with BM-NCC therapy after PNI (peripheral nerve injury).	N = 45 Sex: Male Species: *Sprague Dawley rats* Weight: 180–220 g Age: 8 weeks	Cell viability and proliferation capacity: CCK8 assay (Cell Counting Kit-8). Neuronal morphology: Immunofluorescence, ImageJ software. Bioactive factors: qRT-PCR. Axonal regeneration: Microfluidic devices. Nerve regeneration: Immunohistochemistry, microscopy. Motor and sensory function: Sciatic Functional Index, gait analysis, plantar tests.	The average length of regenerated nerves at 10 days post-surgery was 3.3 mm in the *miR-21-5p* agomir group compared to 1.5 mm in the NC group, representing a 2.2-fold improvement (*p* < 0.001). At 8 weeks, the thickness of the myelin sheath and the diameter of the myelinated axons were significantly greater in the *miR-21-5p* agomir group compared to the NC group (*p* < 0.01). The sciatic function index (SFI) showed a more significant increase in the *miR-21-5p* group at 8 weeks compared to the NC group (*p* < 0.001), indicating better functional recovery. The compound muscle action potential (CMAP) amplitude and motor conduction velocity (MCV) were significantly higher in the *miR-21-5p* group at 8 weeks compared to the NC group (*p* < 0.01). A higher percentage of sensory and motor neurons marked with *FG* (FluoroGold) was observed in the *miR-21-5p* group compared to the NC group at 8 weeks (*p* < 0.001), indicating greater neuronal regeneration. The wet weight ratio of the injured muscles to non-injured muscles was significantly greater in the *miR-21-5p* group than in the NC group (*p* < 0.05), and there was a significant increase in the muscle fiber cross-sectional area (*p* < 0.01).	
Liu et al. [48], 2019	Design: Experimental longitudinal quantitative. Objective: To investigate the effect of mesenchymal stem cell-derived exosomes on nerve regeneration in a nerve injury model.	N = 20 Sex: Female Species: *Sprague Dawley rats* Weight: 170–220 g Age: Adults	Functional recovery: BBB locomotion scale. Nerve regeneration: Nissl staining. Apoptosis: TUNEL assay. Neurotrophic factors: Western blot. Cell migration: Transwell and scratch assay.	Incubation with BMSC-Exos (100 μg/mL) significantly increased the proliferation of human umbilical vein endothelial cells (HUVEC) compared to the control group (PBS) (* *p* < 0.05). BMSC-Exos improved tube formation in HUVECs at 6 h, showing a significant increase in total tube length and branching points compared to the PBS group (* *p* < 0.05). Treatment with BMSC-Exos significantly reduced lipopolysaccharide (LPS)-induced nitric oxide production in microglia, compared to the LPS group (*p* < 0.05). The application of BMSC-Exos significantly decreased the number of TUNEL-positive (apoptotic) neurons treated with glutamate (GLU) compared to the GLU-only group (*p* < 0.05). Rats treated with BMSC-Exos showed significant improvement in the BBB locomotion score compared to the SCI group from the first to the fourth week post-trauma (*p* < 0.05). The administration of BMSCs-Exos significantly reduced the volume of spinal cord injury compared to the LME group (*p* < 0.05). The number of TUNEL-positive cells was dramatically reduced in the BMSCs-Exos group compared to the SCI group on day 1 post-injury (*p* < 0.05). The expression of *NF200*, a neuronal damage marker, was significantly higher in the BMSC-Exos group compared to the LME group on days 1 and 28 post-injury (*p* < 0.05). The number of blood vessels in the injured tissue was significantly greater in the BMSC-Exos group compared to the LME group on day 28 (*p* < 0.05), along with an increase in *VEGF* expression (*p* < 0.05). The deposition of chondroitin sulfate proteoglycans (CSPG) was significantly lower in the BMSC-Exos group compared to the LME group (*p* < 0.05).	
Rao et al. [49], 2019	Design: Experimental longitudinal quantitative. Objective: To evaluate the effectiveness of a biodegradable chitin conduit combined with mesenchymal stem cell-derived exosomes for promoting regeneration of a 10 mm sciatic nerve defect in rats.	N = 24 Species: *Sprague Dawley rats* Weight: 200–220 g Age: 8 weeks	Axonal regeneration and nerve morphology: Transmission electron microscopy (TEM), light microscopy, Image-Pro Plus software (7.1 version). Motor function: CatWalk XT 9.0, Sciatic Functional Index (SFI). Electrophysiological function of the regenerated nerve: Medlec Synergy. Effectiveness of the exosome: Cell proliferation assay (CCK), immunofluorescence. Muscle fiber remodeling: Gastrocnemius muscle weighing, Masson’s trichrome staining.	GMSC-derived exosomes promoted greater Schwann cell proliferation compared to the control group, with a statistically significant difference on day 5 (*p* < 0.01). Treatment with GMSC-derived exosomes significantly increased the length of DRG neurites after 5 days of culture, showing a statistically significant difference compared to the control group (*p* < 0.01). Administration of GMSC-derived exosomes resulted in a significantly greater number of myelinated nerve fibers compared to the control group (*p* < 0.05). Regenerated nerve fibers in the exosome-treated group had a significantly larger diameter compared to the control group (*p* < 0.05). The thickness of the myelin sheath of nerve fibers in the exosome-treated group was significantly greater than in the control group (*p* < 0.05). At 8 weeks post-surgery, the Sciatic Functional Index (SFI) in the exosome group significantly improved compared to the control group (*p* < 0.05). At 12 weeks, the SFI of the exosome-treated group was comparable to the autograft group and significantly better than the empty conduit group (*p* < 0.05).	
Chen et al. [50], 2019	Design: Experimental longitudinal quantitative.Objective: To evaluate the effects of adipose-derived mesenchymal stem cell exosomes (ASC-Exos) on nerve regeneration, specifically in a sciatic nerve injury model in rats.	N = 28 Sex: Males Species: *Sprague Dawley rats* (SD) Weight: 180–220 g Age: Adults	Proliferation of Schwann cells: EdU Apollo 567 Kit. Migration of Schwann cells: Transwell chambers. Growth of neurites in dorsal root ganglion (DRG) neurons: Microscopy and Image J software. Myelin segments: Immunostaining for myelin basic protein (*MBP*) and Image J analysis. Neurotrophic factors (*BDNF* and *NGF*): ELISA kits. Gene expression of SCs: qRT-PCR. Nerve regeneration: Histological analysis with toluidine blue staining and antibodies against NF-H (Neurofilament-H) and *P0* (Myelin Zero Protein). Gastrocnemius muscle weight and area: Image J software and weight ratio calculation.	ASC-Exos increased SC proliferation over 72 h, with a significant increase in EdU-positive cell numbers (*p* < 0.05). Schwann cell migration was 3.02 times higher with 20 µg/mL of ASC-Exos compared to the PBS control group (*p* < 0.001), suggesting that exosomes enhance SCs’ ability to migrate to regeneration areas. In a co-culture model of neurons and SCs, treatment with 20 µg/mL of ASC-Exos significantly increased myelination, as indicated by *MBP* expression (*p* < 0.01), suggesting improved SC capacity for remyelination. In the sciatic nerve regeneration model, ASC-Exos treated groups showed a significant increase in the number of regenerated axons and myelin areas at 4 and 8 weeks post-surgery (*p* < 0.01). Schwann cells treated with ASC-Exos produced significantly higher levels of *NGF* and *BDNF* on days 1, 3, and 6 (*p* < 0.001), indicating that ASC-Exos promotes a favorable environment for nerve regeneration through prolonged secretion of neurotrophic factors. Neurites from DRG treated with ASC-Exos were significantly longer, reaching 119.9 ± 4.292 µm compared to 87.63 ± 3.093 µm in the control group (*p* < 0.001).	
Ma et al. [51], 2019	Design: Experimental longitudinal quantitative.Objective: To evaluate the regenerative capacity of extracellular vesicles derived from human umbilical cord mesenchymal stem cells (hUCMSC-EV) for regenerating peripheral nerves, specifically in a rat sciatic nerve injury model.	N = 48 Species: *Sprague Dawley* Weight: 220–230 gAge: 3–4 weeks	Nerve functionality: Sciatic Functional index (SFI). Muscle weight: Electronic balance. Nerve regeneration: Hematoxylin and eosin (H&E) staining, immunofluorescence. Myelinated axons: Wide-field microscopy. Inflammatory markers: Immunohistochemical analysis. Extracellular vesicles: NanoSight system, TEM.	Treatment with hUCMSC-EVs resulted in a significant increase in Sciatic Functional Index (SFI) at 8 weeks post-surgery, showing almost normal functional recovery with significant differences compared to the control group (*p* < 0.05). The regenerated nerve fibers in the hUCMSC-EV treated group had significantly larger diameters compared to the control group at 8 weeks post-injury, with differences of *p* < 0.05. The density of Schwann cell-positive fibers (S-100 marker) was considerably higher in the hUCMSC-EV group compared to the control group, indicating better myelination (*p* < 0.01). Treatment with hUCMSC-EVs led to a significant increase in the number of myelinated axons at 8 weeks post-surgery, showing significant differences from the control group (*p* < 0.01). The wet weight of the gastrocnemius muscle in the hUCMSC-EV treated group was significantly higher than the control group at 8 weeks, indicating better muscle innervation recovery (*p* < 0.05). Injection of hUCMSC-EVs resulted in a significant reduction of pro-inflammatory cytokines (*IL-6* and *IL-1β*) and an increase in the anti-inflammatory cytokine (*IL-10*) compared to the control group at 3 days post-surgery, suggesting positive modulation of the inflammatory response.	
Gärtner et al. [52], 2014	Design: Experimental longitudinal quantitativeObjective: To evaluate the effectiveness of secretomes derived from stem cells in regenerating transected nerves, analyzing both structural regeneration (via histological studies) and functional recovery (through motor tests and nociceptive function assessments) in an animal model.	N = 30 Species: *Wistar rats* Age: 8–12 weeks	MSCs characteristics: Umbilical cord mesenchymal stem cells (UCX): Flow cytometry analysis. Nociceptive function: Withdrawal reflex latency (WRL). Motor deficit: Extensor postural thrust (EPT). Ankle kinematics: Markers and video recordings. Histological analysis: Histology with toluidine blue and H&E staining. Fiber analysis: Stereological analysis of histological images.	More than 95% of UCX^®^ cells expressed MSC markers (CD44, CD73, CD90, CD105) according to ISCT criteria, confirming their regenerative potential. In the withdrawal reflex latency (WRL), the UCX^®^ treated group showed improvement compared to the control (End-to-End) in the weeks following the intervention, with a significant difference of *p* = 0.000 in week 3. In the extensor postural thrust (EPT) assessment, the End-to-EndUCX group showed improvement in motor response, achieving a value of 0.82 ± 0.07 in week 20 compared to the higher motor deficit values observed in groups treated with Floseal^®^ (0.87 ± 0.03) and UCX^®^ alone (0.86 ± 0.04). The fiber density in the End-to-EndFlosealUCX group was 26,009 ± 6512 N/mm^2^, representing a 64% increase compared to the End-to-End group (20,612 ± 1607 N/mm^2^) and was significantly higher (*p* < 0.05) compared to groups treated only with Floseal^®^ (23,900 ± 4291 N/mm^2^) and End-to-EndUCX (28,821 ± 1202 N/mm^2^). The End-to-EndFlosealUCX group showed an axonal diameter of 2.88 ± 0.42 μm, significantly larger (*p* < 0.05) compared to the End-to-EndUCX group (2.42 ± 0.16 μm) and the End-to-EndFloseal group (2.23 ± 0.21 μm). The myelin thickness in the End-to-EndFlosealUCX group was 0.85 ± 0.05 μm, superior to the thickness in the End-to-End group (0.58 ± 0.03 μm) and End-to-EndUCX (0.77 ± 0.06 μm).	

**Table 3:** Results of methodological quality assessment of the studies.

Study/Autor	JBI—Joanna Briggs Institute	Q1	Q2	Q3	Q4	Q5	Q6	Q7	Q8	Q9	Q10	Q11	Q12	Q13
Chen et al. [40], 2024	10/13	+	-	+	-	-	-	+	+	+	+	+	+	+
Fu et al. [41], 2019	13/13	+	+	+	+	+	+	+	+	+	+	+	+	+
Yu et al. [42], 2021	13/13	+	+	+	+	+	+	+	+	+	+	+	+	+
Prautsch et al. [43], 2020	9/13	-	-	+	+	+	+	+	+	-	+	+	+	-
Alvites et al. [44], 2022	11/13	-	-	+	+	+	+	+	+	+	+	+	+	+
Margiana et al. [45], 2019	9/13	+	-	+	-	-	-	+	+	+	+	+	+	+
Raoofi et al. [46], 2021	11/13	+	-	+	+	+	+	+	-	+	+	+	+	+
Cong et al. [47], 2025	13/13	+	+	+	+	+	+	+	+	+	+	+	+	+
Liu et al. [48], 2019	13/13	+	+	+	+	+	+	+	+	+	+	+	+	+
Rao et al. [49], 2019	10/13	+	-	+	+	-	-	+	+	+	+	+	+	+
Chen et al. [50], 2019	10/13	+	-	+	+	-	-	+	+	+	+	+	+	+
Ma et al. [51], 2019	11/13	+	-	+	+	+	-	+	+	+	+	+	+	+
Gärtner et al. [52], 2014	12/13	+	+	+	+	-	+	+	+	+	+	+	+	+

## Data Availability

No new data were created or analyzed in this study.
